# Selection of Genes Associated with Variations in the Circle of Willis in Gerbils Using Suppression Subtractive Hybridization

**DOI:** 10.1371/journal.pone.0127355

**Published:** 2015-05-14

**Authors:** Zhenkun Li, Xueyun Huo, Shuangyue Zhang, Jing Lu, Changlong Li, Meng Guo, Rui Fu, Zhengming He, Xiaoyan Du, Zhenwen Chen

**Affiliations:** 1 Department of Laboratory Animal Science, School of Basic Medical Science, Capital Medical University, Beijing 100069, China; 2 Institute for Laboratory Animal Resources, National Institutes for Food and Drug Control, Beijing 100050, China; Goethe University, GERMANY

## Abstract

Deformities in the Circle of Willis (CoW) can significantly increase the risk of cerebrovascular disease in humans. However, the molecular mechanisms underlying these deformities have not been understood. Based on our previous studies, variations in the CoW of gerbils are hereditary. A normal CoW is observed in approximately 60% of gerbils, a percentage that also applies to humans. Thus, gerbil is an ideal experimental model for studying variations in the CoW. To study the mechanisms underlying these variations, we selected genes associated with different types of the CoW using suppression subtractive hybridization (SSH). After evaluating the efficiency of SSH using quantitative real-time polymerase chain reaction (qPCR) on subtracted and unsubtracted cDNA and Southern blotting on SSH PCR products, 12 SSH libraries were established. We identified 4 genes (*CST3*, *GNAS*, *GPx4* and *PFN2*) associated with variations in the CoW. These genes were identified with qPCR and Western blotting using 70 expressed sequence tags from the SSH libraries. Cloning and sequencing allowed us to demonstrate that the 4 genes were closely related to mouse genes. We may assume that these 4 genes play an important role in the development of variations in the CoW. This study provides a foundation for further research of genes related to development of variations in the CoW and the mechanisms of dysmorphosis of cerebral vessels.

## Introduction

Cerebrovascular disease is a leading cause of disability, and one of the three major causes of death along with heart diseases and malignant tumors [1]. Data published in the China Health Statistical Yearbook (2010) showed that cerebrovascular disease was the third cause of death among urban residents and the first among rural residents. It has been suggested that cerebrovascular disease is a secondary factor affecting life expectancy in China [2]. Additionally, cerebrovascular disease was ranked among the top 4 causes of death in the United States [3]. The Circle of Willis (CoW), or Willis’ Circle, is a primary arterial collateral structure interconnecting hemispheric circulation within the brain, and it is composed of the anterior cerebral artery (ACA), anterior communicating artery (ACoA), internal carotid artery (ICA), posterior cerebral artery (PCA), and posterior communicating artery (PCoA) [4]. The CoW is related to various cerebrovascular diseases. Studies have shown that a variety of CoW deformities occur in humans; only 45.2% of the population has a normal CoW structure, while the remaining 54.8% has variations in the ACoA and PCoA, and subsequently are at increased risk of cerebrovascular disease [5]. Eighty-five percent of saccular aneurysms occur at the polygon of the CoW [6], and the incidence of ruptured aneurysms in patients with anatomical variation of the CoW can be as high as 75.7% [7]. In addition, variation in the CoW is a risk factor for ischemia and stroke in humans [8] and is significantly linked to intracerebral hemorrhage in ischemic stroke patients after intravenous thrombolysis [9]. Furthermore, variations in the CoW can affect the symptoms and prognosis of cerebral ischemia caused by internal carotid artery occlusion [10].

Elucidating the molecular mechanisms causing variations in the CoW is important for predicting and evaluating cerebrovascular disease risk. In studies of the cerebrovascular diseases, the Mongolian gerbil has been used as a model animal for cerebral ischemia, because of malformations in the CoW observed in this species [11]. Both the PCoA and ACoA of the CoW in gerbils express different types of variations. We identified these variations based on the completeness of the PCoA and ACoA and classified them into 8 categories: Type A-I, both the PCoA and ACoA are complete; Type A-II, the PCoA is complete, but the ACoA is missing; Type A-III, the PCoA is complete, but the left branch of the ACA is small or missing; Type A-IV, the PCoA is complete, but the right branch of the ACA is small or missing; Type B-I, the PCoA is missing and the ACoA is complete; Type B-II, both the PCoA and ACoA are missing; Type B-III, the PCoA is missing and the left branch of the ACA is small or missing; and Type B-IV, the PCoA is missing and the right branch of the ACA is small or missing [12]. The percentage of animals with a normal CoW is about 60%, which also applies to humans (approximately 42–52% in the Western populations and 27% in the Chinese population) [13]. Mongolian gerbils exhibit congenital malformations of the CoW, making them an ideal experimental model for cerebral ischemia research. Moreover, our previous research showed that variations in the CoW of gerbils are hereditary [12], and an inbred strain was developed to provide a genetically consistent animal model for exploring the molecular mechanisms contributing to variations in the CoW. We explored genes that might determine different phenotypes of the CoW at the expression level using suppression subtractive hybridization (SSH) in the inbred strain of gerbils. SSH is used to study the expression levels of genes in different physiological states or different growth stages. This method greatly reduces the false-positive rate, has high sensitivity, high speed, and high efficiency, and its applications has helped to gradually expand knowledge on the genome of various species [14,15] and the genes related to development and reproduction [16]. In this study, we performed SSH to screen the genes associated with variations in the CoW in F_10_ inbred gerbils.

## Results

### Construction of SSH libraries by various types of the CoW

**Fig 1:**
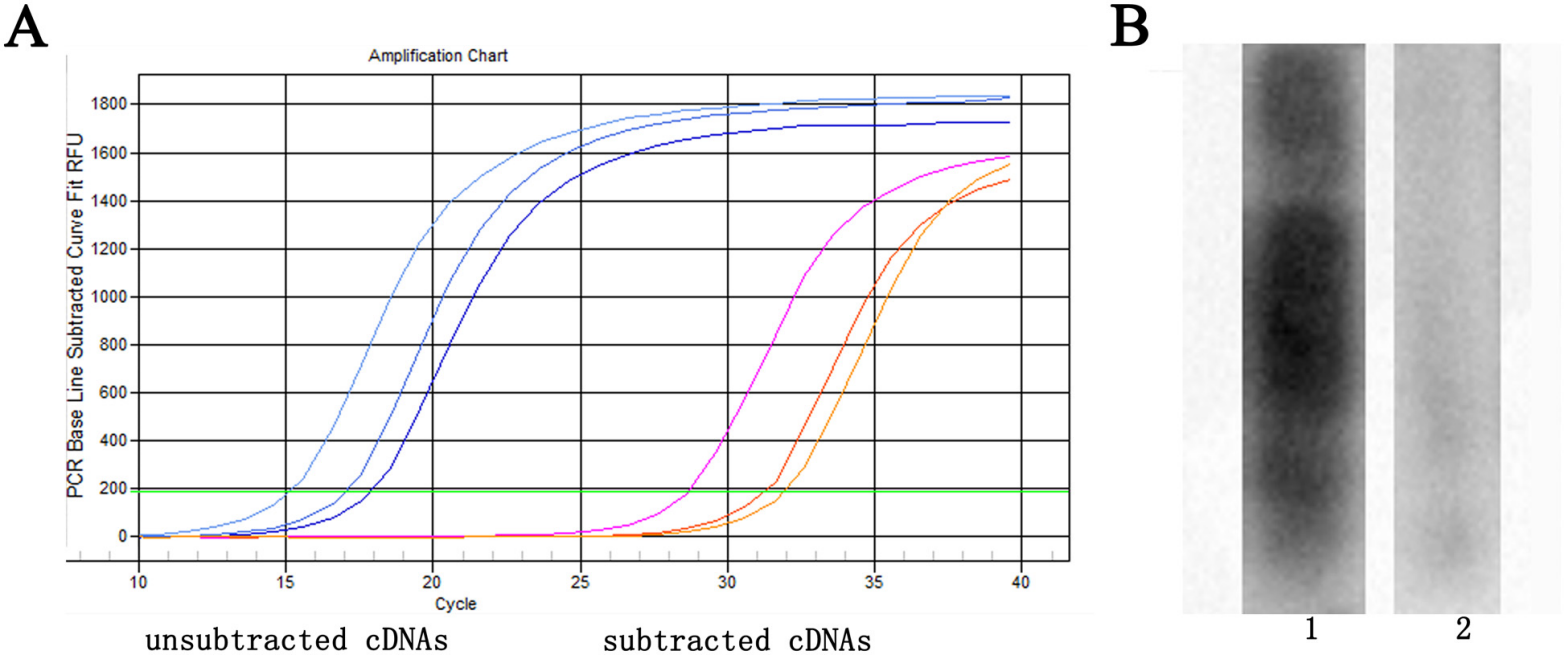
The efficiency of suppression subtractive hybridization (SSH) evaluated by qPCR and Southern blotting. (A) The efficiency was evaluated by qPCR with *β-actin* primers. (B) The efficiency was evaluated by Southern blotting with dig-labeled PCR product hybridized on the PCR products in the same group. The value of grey scanning of the dig-labeled SSH PCR product hybridized with itself (1) (191) is 2-fold higher than that hybridized with the SSH PCR product of another animal in the same SSH group (2) (85).

We developed SSH libraries using 9 brain samples from F_10_ inbred gerbils classified into 4 groups (Group 1–4) and 12 sub-groups based on the CoW type (Table 1). In Group 1, subgroup 1.1 was constructed using the Type B-I sample as the tester and the Type B-II sample as the driver, while subgroup 1.2 was constructed using the Type B-II sample as the tester and the Type B-I sample as the driver. In Group 2, subgroup 2.1 was constructed using the Type B-III sample as the tester and the Type B-I sample as the driver, while subgroup 2.2 was constructed using the Type B-I sample as the tester and the Type B-III sample as the driver. In Group 3, subgroup 3.1 was constructed using the Type B-I sample as the tester and the Type B-IV sample as the driver, while subgroup 3.2 was constructed was constructed using the Type B-IV sample as the tester and the Type B-I sample as the driver. In Group 4, subgroup 4.1 was constructed using the Type A-I sample as the tester and the Type A-IV sample as the driver, while subgroup 4.2 was constructed using the Type A-IV sample as the tester and the Type A-I sample as the driver. Subgroup 4.3 was constructed using the Type A-I sample as the tester and the Type B-I sample as the driver, while subgroup 4.4 was constructed using the Type B-I sample as the tester and the Type A-I sample as the driver. Subgroup 4.5 was constructed using the Type A-IV sample as the tester and the Type B-I sample as the driver, while subgroup 4.6 was constructed using the Type B-I sample as the tester and the Type A-IV sample as the driver. Because SSH can make the differential expression genes being enriched while other genes being hybridized, β-actin, as a housekeeping gene, should exist low expression level in the PCR product after subtracted [17]. Besides, the genes after subtracted should have less cDNA fragments in tester and it can be tested by Southern blotting. Therefore, the efficiency of SSH method was evaluated by quantitative real-time polymerase chain reaction (qPCR) with *β-actin* primers on subtracted and unsubtracted cDNA and Southern blotting on SSH PCR products. Typical results of qPCR and Southern blotting are shown in Fig 1. Fig 1A shows the qPCR results of subgroups 2.1, 2.2, and 4.1. The Ct values of the tester and subtracted cDNA, respectively, of subgroup 2.1 were 16.55 and 31.34; subgroup 2.2, 14.59 and 27.46; and subgroup 4.1, 17.25 and 27.86. The C_t_ values of the testers were lower than those of the subtracted cDNA and the expression level of *β-actin* of the unsubtracted cDNA was about 4000 fold higher than that of the subtracted cDNA. Fig 1B shows the Southern blotting results of subgroup 1.1. The value of grey scanning of the dig-labeled SSH PCR product hybridized with itself was different from that hybridized with the SSH PCR product of another animal in the same SSH group and demonstrated we had a fine effect on SSH. The value of grey scanning of the PCR product of animal number 1 (used as probe) was 191, 2-fold higher than that of animal number 2 (85). In summary, these results showed that our SSH method was highly efficient.

**Table 1:** Types of the Circle of Willis (CoW) in gerbils that used for the development of 12 suppression subtractive hybridization (SSH) libraries (Groups 1–4).

	SSH library group and verified sample	Type of the CoW		Number of samples	
SSH library group	Group1.1	Tester	Type B-I	Tester	1
		Driver	Type B-II	Driver	2
	Group 1.2	Tester	Type B-II	Tester	2
		Driver	Type B-I	Driver	1
	Group 2.1	Tester	Type B-III	Tester	3
		Driver	Type B-I	Driver	4
	Group 2.2	Tester	Type B-I	Tester	4
		Driver	Type B-III	Driver	3
	Group 3.1	Tester	Type B-IV	Tester	5
		Driver	Type B-I	Driver	6
	Group 3.2	Tester	Type B-I	Tester	6
		Driver	Type B-IV	Driver	5
	Group 4.1	Tester	Type A-I	Tester	7
		Driver	Type A-IV	Driver	8
	Group 4.2	Tester	Type A-IV	Tester	8
		Driver	Type A-I	Driver	7
	Group 4.3	Tester	Type A-I	Tester	7
		Driver	Type B-I	Driver	9
	Group 4.4	Tester	Type B-I	Tester	9
		Driver	Type A-I	Driver	7
	Group 4.5	Tester	Type A-IV	Tester	8
		Driver	Type B-I	Driver	9
	Group 4.6	Tester	Type B-I	Tester	9
		Driver	Type A-IV	Driver	8
Verified samples	Group A	Type A-I		10/11/12	
	Group B	Type A-IV		13/14/15	
	Group C	Type B-I		16/17/18	
	Group D	Type B-III		19/20/21	
	Group E	Type B-IV		22/23/24	

### Selection of genes associated with variations of the CoW in gerbil SSH libraries

**Fig 2:**
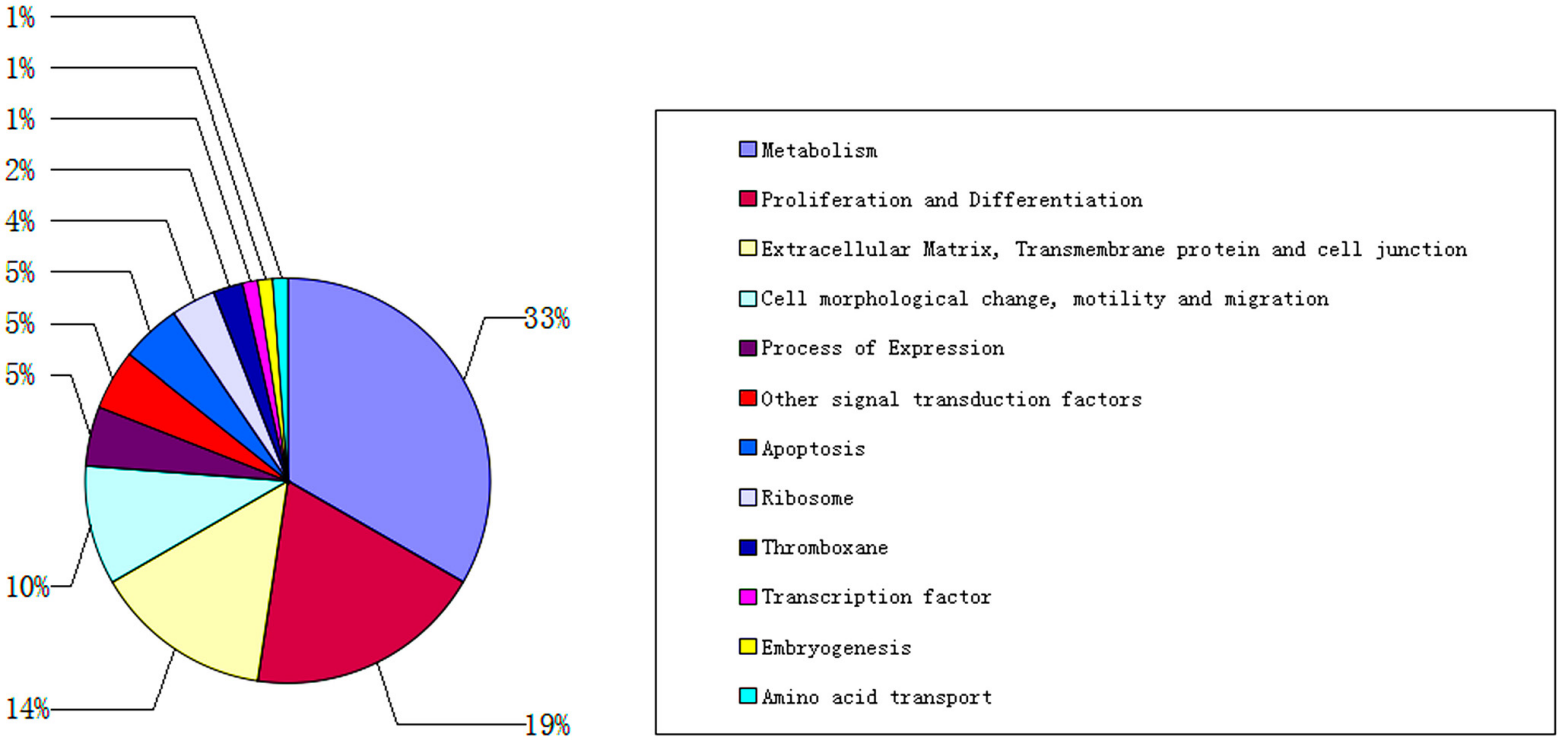
Classification of genes from the suppression subtractive hybridization (SSH) libraries. We identified 304 sequences from 12 SSH libraries. After performing Basic Local Alignment Search Tool (BLAST) analysis, we obtained 84 genes with available information on their function in previous studies or the Mouse Genome Informatics database. These 84 genes were divided into 12 groups according to their functions.

We sequenced approximately 900 positive clones and identified 304 cDNA sequences from 12 SSH libraries. The size of cDNA sequences within the libraries ranged from 50 to 600 bp. Sequence analysis using Basic Local Alignment Search Tool (BLAST) revealed that 84 out of 304 genes (27.63%) had previously been identified and information on their function was available on the Mouse Genomic Database as well as in previously reported studies. All of the expressed sequence tags (ESTs) of gerbil were submitted to GenBank and their IDs and names are presented in S1 Table. Based on similarities to sequences found in the National Center for Biotechnology Information (NCBI) databases, 84 genes including ESTs were divided into 12 functional categories (Fig 2). Metabolism-related genes formed the largest category, representing 33% of the total identified genes. Proliferation or differentiation-related genes formed the second largest category (19% of the total identified genes) and genes related to the extracellular matrix, transmembrane proteins, or cell junctions formed the third largest category (14% of the total identified genes). Ten percent of total identified genes were related to cell morphology changes, motility, or migration and 5% of total identified genes were related to expression, apoptosis, and other signal transduction factors. Four percent of total identified genes were related to ribosome function and 2% of total identified genes were related to thromboxane. Finally, 1% of total identified genes were related to transcription factors, embryogenesis, and amino acid transport. We found that 16 genes were related to vasculogenesis or angiogenesis by reviewing related literature and the Mouse Genome Informatics database.

### Verification of 16 genes most likely related to vasculogenesis or angiogenesis with qPCR

**Fig 3:**
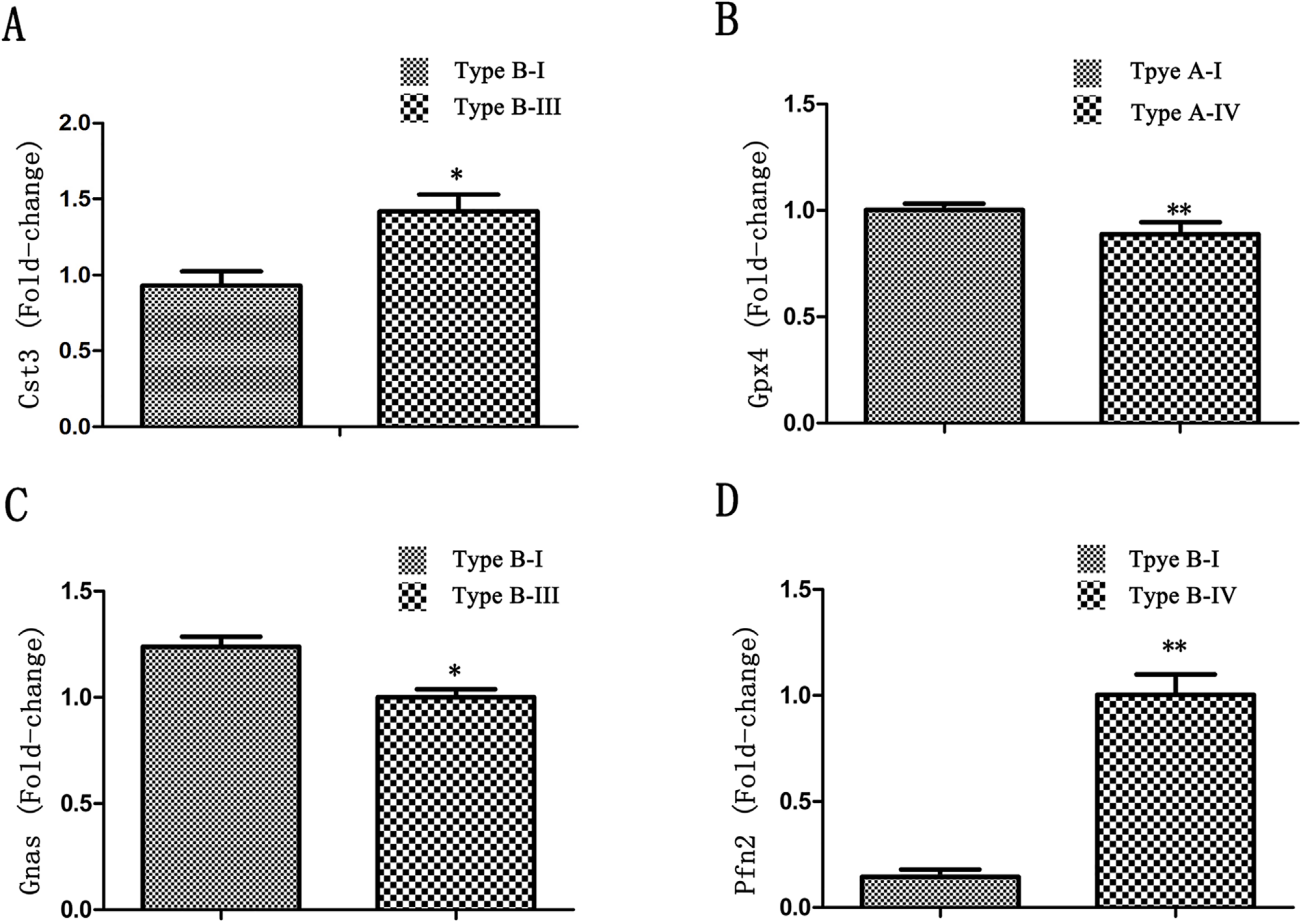
Relative expression of 4 genes (*CST3*, *GNAS*, *GPx4*, and *PFN2*) with significantly different expression levels between different types of the Circle of Willis. (A) The relative expression levels of CST3 between the Type B-I and Type B-III. (B) The relative expression levels of GNAS between the Type B-I and Type B-III. (C) The relative expression of levels GPx4 between the Type A-IV and Type A-I. (D) The relative expression levels of PFN2 between the Type B-I and Type B-IV. * p <0.05, ** p <0.01.

We further characterized the 16 genes most likely related to vascular development or angiogenesis with qPCR. Based on Table 1, the 16 genes were derived from 7 SSH subgroups and we used the samples from the corresponding CoW types to identify these genes. The results showed that 4 out of 16 genes (25.0%) had significantly different expression level from the corresponding types of the CoW within same SSH group, and CoW patterns of the higher expression sample were in accordance with those of the tester in the corresponding SSH subgroup (Fig 3). These 4 genes were cysteine proteinase inhibitor *cystatin C* (*CST3*), *guanine nucleotide binding protein alpha stimulating* (*GNAS*), *glutathione peroxidase 4* (*GPx4*), and *profilin 2* (*PFN2*). *CST3* was identified from subgroup 2.1 and its relative expression level in the Type B-I was significantly higher than that in the Type B-III (p = 0.042, ≤0.05). *GNAS* was identified from subgroup 2.2 and its relative expression level in the Type B-I was significantly higher than that in the Type B-III (p = 0.032, ≤ 0.05). *GPx4* and *PFN2* were both identified from subgroup 4.1. The relative expression level of GPx4 in the Type A-I was significantly higher than that in the Type A-IV (p = 0.003, ≤ 0.01) and of PFN2 in the Type B-I was significant higher than that in the Type B-IV (p = 0.001, ≤ 0.01). These results indicated that these 4 genes might be related to the CoW patterns in gerbils.

### Molecular cloning of the coding sequence (CDS) of *PFN2*, *GPx4*, *GNAS*, and *CST3* and homology analysis with other species by DNA Star

**Fig 4:**
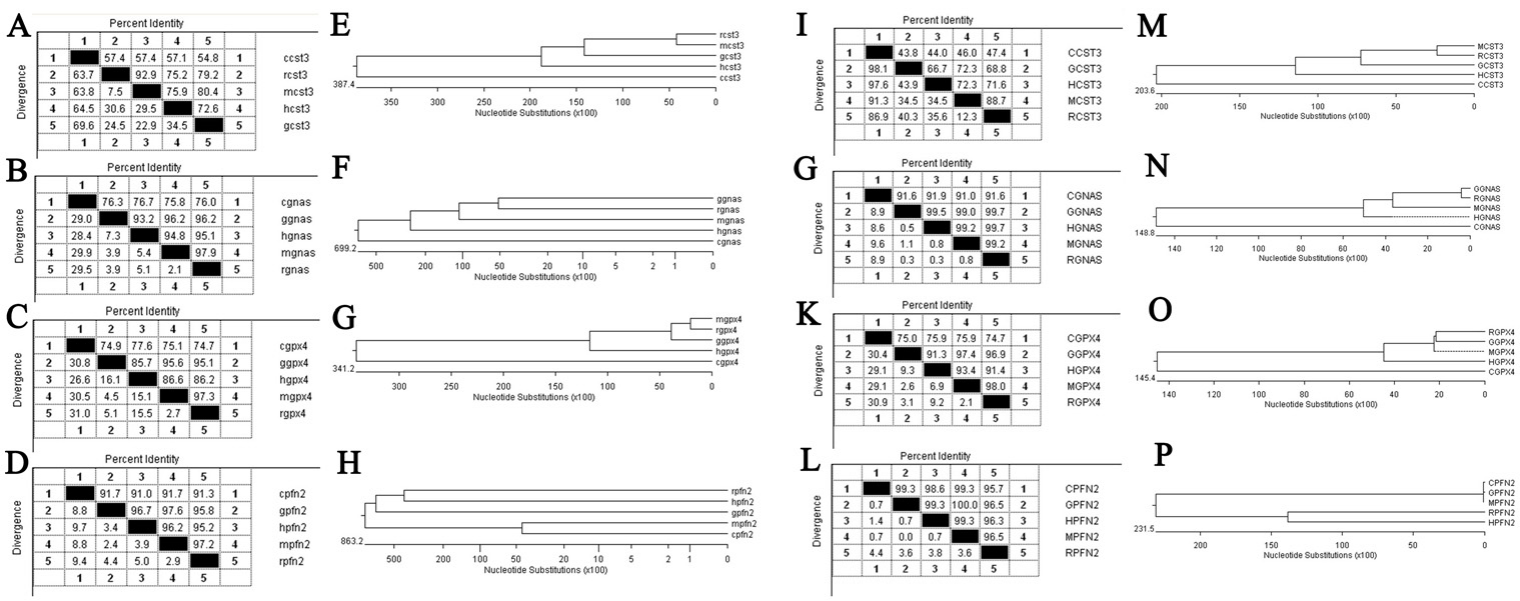
Analysis of open reading frames (ORFs) of *CST3*, *GNAS*, *GPx4*, and *PFN2* with DNAStar. We analyzed the obtained sequences with the ORFs of Gallus gallus (chicken), Mus musculus (mouse), and Rattus norvegicus (rat), and *Homo sapiens* (human). We aligned the sequence distances and drew the phylogenetic tree to analyze their homology. A–D show the results of the DNA sequence distances analysis and E–H show their phylogenetic tree. I–L show the results of the protein sequence distance analysis and M–P show their phylogenetic tree. c/C- *Gallus gallus*, g/G- Gerbils, h/H- *Homo sapiens*, r/R- *Rattus norvegicus*, m- *Mus musculus*.

We obtained the CDS of the 4 genes *PFN2*, *GPx4*, *GNAS*, and *CST3* with primers that we designed and presented in Table 2. The sequences of these 4 genes were submitted to GenBank along with their accession numbers (Table 2). We analyzed the CDSs and compared them against 3 common experimental animals [*Gallus gallus* (chicken), *Mus musculus* (mouse), and *Rattus norvegicus* (rat)] and *Homo sapiens* (human). We performed sequence distance analysis and drew the phylogenetic tree to analyze their homology. We found that the sequences of the CDSs of the 4 genes in gerbils were similar to the sequences of other species. *PFN2* of gerbils showed greater similarity with that of other species (91.7%, 97.6%, 95.8%, and 96.7% with that of chicken, mouse, rat, and human, respectively) than the other 3 genes, while *CST3* of gerbils had the lowest similarity with that of other species (54.8%, 80.4%, 79.2% and 72.6% with that of chicken, mouse, rat, and human, respectively) (Fig 4). The protein products of the 4 genes in gerbils also shared common amino-acid sequences with the other 3 species and human (the highest was for PFN2 and lowest for CST3). Based on the results, we performed Western blotting using mouse antibodies to analyze the proteins encoded by these 4 genes in the gerbil brain.

**Table 2:** Accession number, primer sequences, annealing temperatures, and length of PCR products used for PCR cloning of *CST3*, *GNAS*, *GPx4* and *PFN2*.

Genes	Accession number	Primer	Sequence	Annealing temperature (°C)	Product length (bp)
*CST3*	KM517575	Forward Primer	AAAAGTCGCACGGAGTAGCA	58	630
		Reverse Primer	CCTGCAGCAGCTCCTTTACT		
*GNAS*	KM517576	Forward Primer	GCTGCCTCGGCAACAGTAA	58	1389
		Reverse Primer	CTTTTTCGTCTGTAGGCCGC		
*GPx4*	KM517578	Forward Primer	ACCAATAAGAGACGTCAGTGGG	58	802
		Reverse Primer	TGGTTTTCAGGCAGACCTTCA		
*PFN2*	KM517577	Forward Primer	TAACCTGATGTGCGATGGCT	58	816
		Reverse Primer	CACCGGCACCTTGCTTACTA		

### Verification of qPCR-positive genes with Western blotting

**Fig 5:**
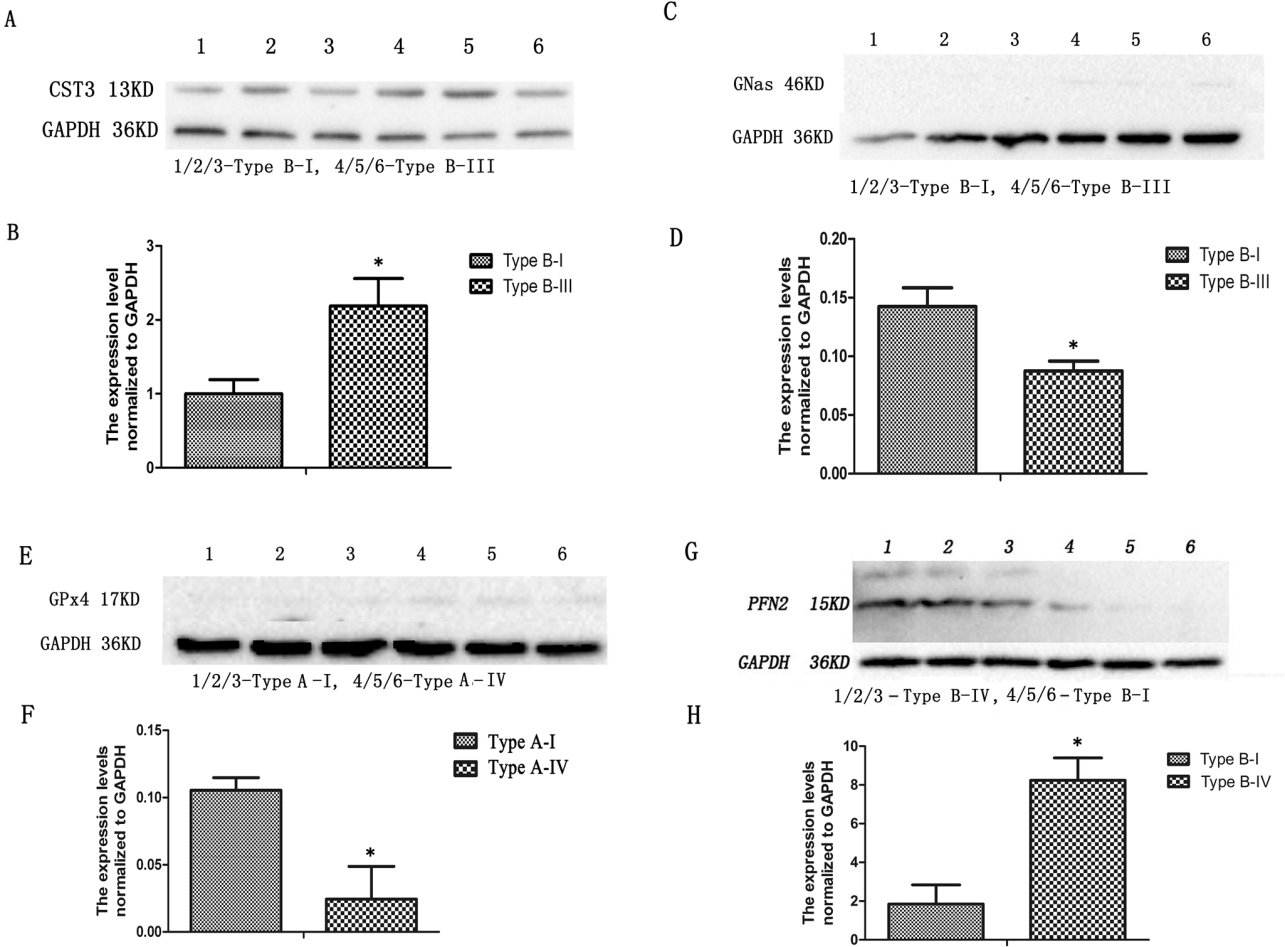
Verification of genes identified from the suppression subtractive hybridization (SSH) libraries with Western blotting. (A) The protein level of CST3 between the Type B-I and Type B-III. (B) The semi-quantitative protein level of CST3. (C) The protein level of GNAS between the Type B-I and Type B-III. (D) The semi-quantitative protein level of GNAS. (E) The protein level of GPx4 between the Type A-I and Type A-IV. (F) The semi-quantitative protein level of GPx4. (G) The protein level of PFN2 between Type B-I and Type B-IV. (H) The semi-quantitative protein level of PFN2. * p <0.05.

We further verified the 4 genes related to vasculogenesis or angiogenesis by Western blotting using glyceraldehyde 3-phosphate dehydrogenase (GAPDH) as an internal control. The results (Fig 5) demonstrated that the expression level of CST3 in the Type B-I was significantly higher than that in the Type B-III (p = 0.048, ≤ 0.05). The expression level of GNAS in the Type B-I was significantly higher than that in the Type B-III (p = 0.039, ≤ 0.05). The expression level of GPx4 was significantly higher in the Type A-I than that in the Type A-III (p = 0.037, ≤ 0.05). The expression level of PFN2 in the Type B-I was significantly higher than that in the Type B-IV (p = 0.014, ≤ 0.05). These results were consistent with those obtained from qPCR and further confirmed that the 4 genes were associated with variations of the CoW in gerbils.

## Discussion

SSH is one of the most effective methods to identify differentially expressed genes from various samples [17], and it has been widely used in multiple species and tissues [18–20]. We used samples obtained from inbred gerbils to perform SSH and identify genes related to different types of the CoW. Every pair of tester and driver cDNA originated from the same litter to reduce genetic diversity as much as possible. All the animals that we used were from F_10_ and inbred by sister and brother mating, which minimized genetic diversity while maintaining differences in the phenotypes of different patterns of the CoW.

We applied SSH to determine the genes that were related to variations in the CoW. From 12 SSH libraries, we identified 304 gene sequences in which 23% were ESTs. The percentage was less than that in previous reports [21,22], which might be attributed that in this study we used inbred gerbils with high genetic consistency. Most of the identified genes were associated with proliferation, differentiation, migration, and apoptosis, all of which may influence vascular development. Using the ESTs, we identified 4 genes (*PFN2*, *GPx4*, *GNAS*, and *CST3*) associated with variations in the CoW in gerbils.

We cloned the 4 genes and identified their CDSs. After the analysis of CDSs and amino-acid sequences of the 4 genes, we found that the sequences from gerbils had a high level of homology with sequences of other species. Hence, the Western blotting results using mouse antibodies were reliable. PFN2 showed the greatest homology between gerbils and the other study species, while CST3 had the least amount of homology. Furthermore, the percent identity of PFN2 between humans and gerbils was 96.7%, which was higher than that between humans and rats (95.2%) and between humans and mice (96.2%), suggesting that the gerbil is a better animal model for studying the function of PFN2 in humans. It was shown that the 4 genes shared a good homology among the 5 species, while the best was between gerbils, mice, and rats. Our previous research showed a higher percentage of cross-amplification between the gerbil and mouse primers than that between the gerbil and rat primers, suggesting that the gerbil may be more genetically similar to the mouse than to the rat [23], which was also verified in the present study.

CST3 is the most crucial extracellular inhibitor of many cysteine proteinases, and an imbalance between cysteine proteinases and cystatins can lead to connective tissue remodeling [24]. It has been shown that the expression of vascular endothelial growth factor (VEGF) is correlated with CST3 in patients with esophageal carcinoma [25]. The authors suggested that this correlation might be due to the diminishment of renal clearance of the low-weight VEGF and CST3 or the alteration of the glomerular filtration ratio caused by the increased endothelial cell proliferation in the glomeruli induced by increased amounts of VEGF, rather than the direct or indirect interaction between the two proteins. CST3 is an inhibitor of cathepsins, which inhibit the tubule formation of endothelial cells [26] and show antiangiogenic characteristics in vitro [27]. It has been confirmed that CST3 is degraded by matrix metalloproteinase-2 (MMP-2), which is a potent pluripotential angiogenic stimulator. MMP-2 degrades and inactivates VEGF-binding inhibitory proteins without degrading or inactivating VEGF [28]. Hence, CST3 and VEGF should have reverse functions. Alternatively, CST3 inhibits vascular development, which is consistent with our findings that the expression level of CST3 is higher in brains with a complete ACoA than in those where the ACoA is small on the left side. The complex locus, *GNAS*, gives rise to multiple gene products [29], mediating the actions of endogenous molecules through the generation of intracellular cyclic AMP (cAMP) [30]. It has been reported that cAMP analogs (8-Bromo-cAMP) and forskolin (an adenylate cyclase activator) decrease transforming growth factor beta 1 (TGFβ1)-induced angiogenesis in vitro in mouse endothelial cell lines and in primary cultures of human umbilical vein endothelial cells [31]. Alternatively, Thaker et al. identified the β-adrenergic activation of the cAMP—PKA signaling pathway as one of the mechanisms that enhances tumor angiogenesis in vivo [32]. The mechanism of *GNAS* affects the cerebral vascular development probably through this pathway. GPx4 is an important intracellular antioxidant enzyme that reduces the level of phospholipid hydroperoxides [33]. Schneider et al. showed that GPx4 plays an important role in angiogenesis and vessel maturation by regulating the activity of 12/15-lipoxygenase [34] and is also associated with VEGF [34]. The expression of GPx4 in the right side of the incomplete ACoA and the complete ACoA was significantly different. Previous studies have shown that the migration of endothelial progenitor or endothelial cells occurs in both vasculogenesis and angiogenesis, requiring multiple cellular processes including polymerization and depolymerization of the actin cytoskeleton regulated by actin-binding proteins [35]. Among the actin-binding proteins, PFNs are the pivotal actin polymerization regulators. PFNs have 4 isoforms [36] of which PFN1 and PFN2 play non-redundant roles in the non-canonical Wnt signaling pathway as effectors for disheveled-associated activation of morphogenesis 1 (DAAM1) in vertebrate gastrulation of *Xenopus* [37]. We hypothesize that PFN2 may affect vasculogenesis or angiogenesis during vertebrate gastrulation in gerbils. This was supported by our results that the expression level of PFN2 was higher in the right side of the incomplete ACoA than that in the complete ACoA.

The 4 genes identified from the SSH libraries and validated by qPCR might affect vascular development independently, congruently, or by interacting with other genes related to vascular development. Many genes identified in this study are poorly understood and may also influence the variations in the CoW. In conclusion, we performed SSH using inbred gerbils and identified 4 genes (*CST3*, *GNAS*, *GPx4* and *PFN2*) associated with variations in the CoW.

## Materials and Methods

### Ethics statement

All the experiments and animal procedures were conducted in accordance with the Guideline of the Capital Medical University Animal Experiments and the Experimental Animals Management Committee. The protocol was approved by the Animal Experiments and Experimental Animal Welfare Committee of Capital Medical University (Permit Number: 2011-X-009).

### Animal material

In this study, the brains of 24 adult animals (age 6–10 months) were screened; 9 animals were used to build SSH libraries, and the other 15 were used to verify the selected genes. First, the animals were given an overdose of pentobarbital and euthanized. The brains were removed, the PCoA and ACoA were evaluated using a Leica EZ4 dissecting microscope (Leica, USA), and photos were taken. We retrieved 24 brain samples, which were grouped according to the type of the CoW and stored in liquid nitrogen until use (Table 1).

### Construction of SSH libraries

We used each pair of samples as indicated in Table 1 to establish 2 or 6 SSH libraries for each group (Table 1). Total RNA was extracted from frozen tissues using TRIzol reagent (Tiangen, China). Purification of mRNA from identical amounts of total RNA (200 μg) was performed using Purification of poly(A) RNA kit (Macherey-Nagel, Germany) following manufacturer’s instructions. We synthesized cDNA using Universal RiboClone cDNA Synthesis System (Promega, USA) following manufacturer’s instructions. SSH was employed to identify distinct genes between various types of the CoW using PCR-Select cDNA Subtraction Kit (Clontech, USA) following the manufacturer’s instructions. Subtractions were performed using a 5-fold excess amount of driver cDNA against tester cDNA. Finally, the subtraction efficiency was evaluated by qPCR with *β-actin* primers on subtracted or unsubtracted cDNA and Southern blotting with dig-labeled SSH PCR product on itself and the SSH PCR product of another animal in the same SSH group. An iQ5 thermal cycler (Bio-Rad, USA) was used to perform qPCR as follows: pre-denaturation at 95°C for 15 min, 40 cycles of denaturation at 95°C for 10 s, annealing and extension at 60°C for 35 s, and 71 cycles of melt-curve analysis at 60°C for 10 s. The processes of Southern blotting were performed using DIG High Prime DNA Labeling and Detection Starter Kit II (Roche, USA). Grey scanning value was obtained using Band Leader 3.0 (copyright of M. Aharoni). The confirmed cDNA with high subtraction efficiency was purified using a Gel purification kit (Axygen, USA), ligated into the pMD19-T Vector (TaKaRa, Japan), transformed into DH5a competent cells (ExCell, China), and maintained at 37°C with shaking (200 rotations min^-1^) overnight followed by plating onto agar plates containing ampicillin, X-gal, and isopropyl-β-d-thiogalactoside (IPTG). Then, we performed DNA sequencing (Ruiboxingke, China) of SSH libraries and BLAST analysis.

### Analysis of expressed genes in different types of the CoW with qPCR

After cDNA sequencing and BLAST analysis, we designed primers (Table 3) for 16 genes that were likely related to vascular development and angiogenesis, in order to analyze the distinct expression of these genes in different types of the CoW using qPCR. An iQ5 thermal cycler (Bio-Rad, USA) was used to perform qPCR as follows: pre-denaturation at 95°C for 15 min, 40 cycles of denaturation at 95°C for 10 s, annealing and extension at 60°C for 35 s, and 71 cycles of melt curve analysis at 60°C for 10 s. The expression levels of gene transcripts were normalized to *β-actin*. Brain tissues from 3 animals were used for the identification of gene expression level of each type of the CoW. A total of 15 adult animal brains were used and their CoW patterns are presented in Table 1. Total RNA was extracted from frozen tissues using TRIzol reagent (Tiangen, China), and the total RNA was treated with DNase I (RNase Free) (Tiangen, China). The first strand of cDNA was synthesized from total RNA using Fast Quant RT Kit (Tiangen, China). Reverse transcription (RT) reaction was performed as follows: 42°C for 15 min followed by 95°C for 3 min.

**Table 3:** Gene names, primer sequences, annealing temperatures, and length of PCR products used for qPCR analysis of 16 genes related to vasculogenesis or angiogenesis.

Gene	Primer	Sequence	Annealing temperature (°C)	Product length (bp)
*β-tubulin*	Forward Primer	CGAACAGATGCTTAACGTCCAA	60	100
	Reverse Primer	GCCCCGAGGTGGAATGTC		
*WNK*	Forward Primer	TTCCCGGACCGTCTCTAATTC	60	126
	Reverse Primer	AAGACATCGTAGACAAAGGACCAACT		
*Catenin beta 1*	Forward Primer	GCAACCCTGAGGAAGAAGATGT	60	104
	Reverse Primer	CCCGTCAATATCAGCTACTTGCT		
*GNAS*	Forward Primer	TCAAGCAGGCCGACTACGT	60	100
	Reverse Primer	TTGACTTTGTCCACCTGGAACTT		
*Basigin*	Forward Primer	ACAGCAGTGGCGTTGACATC	60	105
	Reverse Primer	CTGCGTCCACTATGTACTTCGTATG		
*Cofilin 2*	Forward Primer	GCTCCGTGTGCGCTCTCT	60	109
	Reverse Primer	CCAGTAGAATCAAGTCCAGTTTTGC		
*CST3*	Forward Primer	GCGTTGGACTTCGCTGTGA	60	101
	Reverse Primer	CCAGCCACGAGCTGCTTAC		
*GPx4*	Forward Primer	AGGCAGGAGCCAGGAAGTAATC	60	102
	Reverse Primer	GGCATCGTCCCCATTTACAC		
*Id1*	Forward Primer	CGACATGAACGGCTGCTACTC	60	100
	Reverse Primer	GTCGATTACATGCTGCAGGATCT		
*Pkp4*	Forward Primer	CGAGCTTCTAAGGATGGATAATGAC	60	104
	Reverse Primer	CGTATTTACCTATGAGCTCCTTGTTG		
*PFN2*	Forward Primer	AGCAAGGTGCCGGTGTACA	60	100
	Reverse Primer	AACCATGCGCTACAAAAGGAA		
*Tspan7*	Forward Primer	TGCTGGCATTTCTGGATTTGT	60	100
	Reverse Primer	CCTCTCATCATTGCCATTGTAGTT		
*Tnfrsf1b*	Forward Primer	ACACCCTACAAACCGGAACCT	60	104
	Reverse Primer	ACATATTGGCCAGGAGGACACTTA		
*Fzd8*	Forward Primer	GCAGCGGGGACTTAAATGAC	60	106
	Reverse Primer	ACCATTGTCTGGGGTAAGTGC		
*Rtn4*	Forward Primer	CCACCCATTCAGGGCATATT	60	110
	Reverse Primer	TCAATTCTTTTATTGTGCTGTTCACA		
*SC1*	Forward Primer	AACGCGCAAACATCACAAAG	60	100
	Reverse Primer	TTTAAGTCTTGGGTTTGCTTGCT		
*β-actin(Internal control)*	Forward Primer	AGAGGGAAATCGTGCGTGAC	60	137
	Reverse Primer	CAATAGTGATGACCTGGCCGT		

### CDS cloning of verified genes and analysis with DNA Star

Four genes (*CST3*, *GNAS*, *GPx4* and *PFN2*) were confirmed by qPCR and identified as being associated with variations in the CoW. To clone these 4 genes, PCR was performed using full-length cDNA synthesized previously with pfu high-fidelity DNA polymerase that kindly provided by Professor Li. The primers used and the annealing temperatures are presented in Table 2. The experiment was performed on a MyCycler thermal cycler (Bio-Rad, USA) as follows: 95°C for 5 min for pre-denaturation, 30 cycles of 95°C for 30 s, 55°C for 30 s, 72°C for 120 s, and a final extension at 72°C for 5 min. Electrophoresis of PCR products was performed on a 2% agarose gel. DNA sequencing (Ruiboxingke, China) and sequence analysis were performed with DNA Star v 7.1 software (DNA Star, USA).

### Western blotting analysis

After qPCR analysis, the 4 possible CoW variation-related proteins were analyzed by Western blotting. Proteins were extracted from the samples using Proteins Extraction Kit (CWBIO, China) and quantified with BCA-Reagents (CWBIO, China). Protein lysates were subjected to sodium dodecyl sulfate-polyacrylamide gel electrophoresis (SDS-PAGE) at 160 V on 12% gel (CWBIO, China) for 1 h and then transferred to a 0.22 μm nitrocellulose filter membrane (NC) at 200 mA for 3 h. The primary antibodies CST3 and PFN2 (Santa, USA) were diluted 1:100, GNAS and GPx4 (abcam, UK) were diluted 1:2500, and the secondary antibodies were diluted 1:5000. The membranes were washed completely and visualized with enhanced chemiluminescence (ECL) immunoblotting detection reagents (Thermo Fisher Scientific, USA). Semi-quantitative results were normalized to the housekeeping gene *GAPDH* after gray scanning.

### Statistical analysis

Statistical analysis was performed using SPSS 16.0 (SPSS Inc., USA). Comparisons between different groups were conducted using Student’s t-test. Means were considered significant at p < 0.05 and highly significant at p < 0.01.

## Supporting Information

S1 Fig Twelve genes with no significantly different expression levels between the different types of the Circle of Willis. (TIF)

S1 Table The User Id, dbEST Id and GenBank Accn of the EST we got from the SSH libraries. (PDF)
