# Supplementary figures and images for: Selection of Genes Associated with Variations in the Circle of Willis in Gerbils Using Suppression Subtractive Hybridization
Source: PLoS One. 2015 May 14;10(5):e0127355. doi: 10.1371/journal.pone.0127355 (PMC4431780; doi:10.1371/journal.pone.0127355)

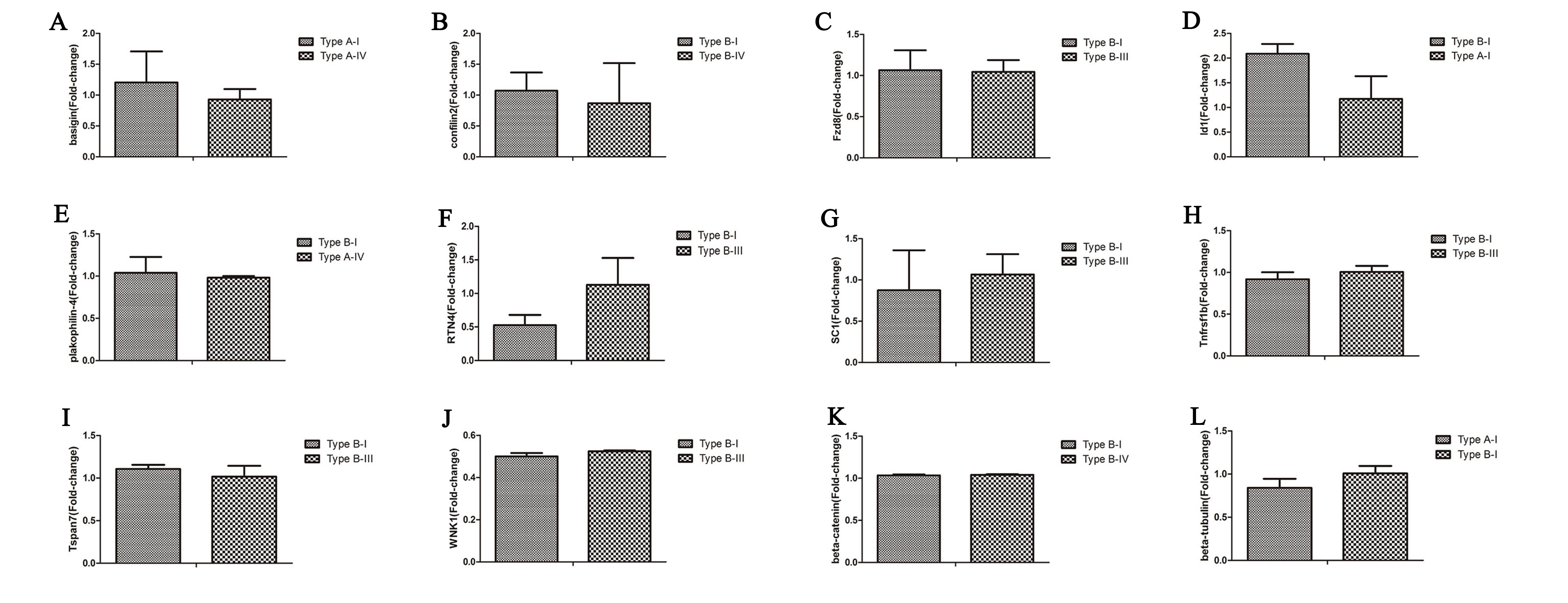

Supplement: S1 Fig — (TIF) [file pone.0127355.s001.tif]
