# Supplementary material for: Selection of Genes Associated with Variations in the Circle of Willis in Gerbils Using Suppression Subtractive Hybridization
Source: PLoS One. 2015 May 14;10(5):e0127355. doi: 10.1371/journal.pone.0127355 (PMC4431780; doi:10.1371/journal.pone.0127355)
Supplement: S1 Table — (PDF) [file pone.0127355.s002.pdf]

User Id	dbEST Id	GenBank Accn
GerbilEST1	79492189	JZ732990
GerbilEST2	79492190	JZ732991
GerbilEST3	79492191	JZ732992
GerbilEST4	79492192	JZ732993
GerbilEST5	79492193	JZ732994
GerbilEST6	79492194	JZ732995
GerbilEST7	79492195	JZ732996
GerbilEST8	79492196	JZ732997
GerbilEST9	79492197	JZ732998
GerbilEST10	79492198	JZ732999
GerbilEST11	79492199	JZ733000
GerbilEST12	79492200	JZ733001
GerbilEST13	79492201	JZ733002
GerbilEST14	79492202	JZ733003
GerbilEST15	79492203	JZ733004
GerbilEST16	79492204	JZ733005
GerbilEST17	79492205	JZ733006
GerbilEST18	79492206	JZ733007
GerbilEST19	79492207	JZ733008
GerbilEST20	79492208	JZ733009
GerbilEST21	79492209	JZ733010
GerbilEST22	79492210	JZ733011
GerbilEST23	79492211	JZ733012
GerbilEST24	79492212	JZ733013
GerbilEST25	79492213	JZ733014
GerbilEST26	79492214	JZ733015
GerbilEST27	79492215	JZ733016
GerbilEST28	79492216	JZ733017
GerbilEST29	79492217	JZ733018
GerbilEST30	79492218	JZ733019
GerbilEST31	79492219	JZ733020
GerbilEST32	79492220	JZ733021
GerbilEST33	79492221	JZ733022
GerbilEST34	79492222	JZ733023
GerbilEST35	79492223	JZ733024
GerbilEST36	79492224	JZ733025
GerbilEST37	79492225	JZ733026
GerbilEST38	79492226	JZ733027
GerbilEST39	79492227	JZ733028
GerbilEST40	79492228	JZ733029
GerbilEST41	79492229	JZ733030
GerbilEST42	79492230	JZ733031

GerbilEST43	79492231	JZ733032
GerbilEST44	79492232	JZ733033
GerbilEST45	79492233	JZ733034
GerbilEST46	79492234	JZ733035
GerbilEST47	79492235	JZ733036
GerbilEST48	79492236	JZ733037
GerbilEST49	79492237	JZ733038
GerbilEST50	79492238	JZ733039
GerbilEST51	79492239	JZ733040
GerbilEST52	79492240	JZ733041
GerbilEST53	79492241	JZ733042
GerbilEST54	79492242	JZ733043
GerbilEST55	79492243	JZ733044
GerbilEST56	79492244	JZ733045
GerbilEST57	79492245	JZ733046
GerbilEST58	79492246	JZ733047
GerbilEST59	79492247	JZ733048
GerbilEST60	79492248	JZ733049
GerbilEST61	79492249	JZ733050
GerbilEST62	79492250	JZ733051
GerbilEST63	79492251	JZ733052
GerbilEST64	79492252	JZ733053
GerbilEST65	79492253	JZ733054
GerbilEST66	79492254	JZ733055
GerbilEST67	79492255	JZ733056
GerbilEST68	79492256	JZ733057
GerbilEST69	79492257	JZ733058
GerbilEST70	79492258	JZ733059
